# Should ECMO be used in cardiogenic shock?

**DOI:** 10.1186/s13054-019-2453-2

**Published:** 2019-05-16

**Authors:** Orhan Gokalp, Koksal Donmez, Hasan Iner, Gamze Gokalp, Yuksel Besir, Nihan Karakas Yesilkaya, Levent Yilik, Ali Gurbuz

**Affiliations:** 1 Faculty of Medicine, Department of Cardiovascular Surgery, Izmir Katip Celebi University, Izmir, Turkey; 2 Department of Cardiovascular Surgery, Izmir Katip Celebi University, Ataturk Education and Research Hospital, Izmir, Turkey; 3 Department of Pediatric Emergency, Tepecik Education and Research Hospital, Izmir, Turkey; 4 Department of Cardiovascular Surgery, Tunceli State Hospital, Tunceli, Turkey

We read the compilation of Hajjar et al. with interest [1]. Cardiogenic shock is the clinical expression of circulatory failure, because of left, right, or biventricular dysfunction. One of the most common applications to eliminate circulatory failure is the extracorporeal membrane oxygenation (ECMO), as stated by the authors. Theoretically, ECMO will provide the necessary circulatory support. However, it is unrealistic to expect ECMO to improve cardiac functions in a patient with cardiogenic shock. A veno-arterial (VA) ECMO that is applied in the case of a cardiogenic shock due to left ventricular (LV) failure is almost impossible to improve LV functions. Because, in order to improve myositis damage in a failing ventricle, the wall tension of the ventricle must be decreased. This is only possible by venting or in other words unloading the failing ventricle. However, it cannot be expected that the VA-ECMO can vent the left ventricle.

On the other hand, in a patient with cardiogenic shock, VA-ECMO, which is administered by peripheral cannulation, will increase the left ventricular afterload and increase stress of an already dysfunctional LV. Although this type of application is usually accompanied with introduction of an intra-aortic balloon pump for reducing tension by a degree; unfortunately, this does not change the fact that ECMO increases the afterload.

In the light of this information, the benefit of VA-ECMO in cardiogenic shock appears to be a more advanced LV support system or bridge to heart transplantation, as the authors suggest. We believe that instead of VA-ECMO, it would be more appropriate to use devices such as TandemHeart or Impella, in which the left ventricle is vented in patients with a higher likelihood of improvement in the left ventricle, such as post-cardiotomy cardiogenic shock, primary percutaneous transcoronary angioplasty, or myocarditis. We think that learning the ideas of the authors on this subject will add value to their study.

## References

[1] Hajjar LA, Teboul JL (2019). Mechanical circulatory support devices for cardiogenic shock: state of the art. Crit Care.

[2] Mebazaa A, Combes A, van Diepen S (2018). Management of cardiogenic shock complicating myocardial infarction. Intensive Care Med.

[3] Guglin Maya, Zucker Mark J., Bazan Vanessa M., Bozkurt Biykem, El Banayosy Aly, Estep Jerry D., Gurley John, Nelson Karl, Malyala Rajasekhar, Panjrath Gurusher S., Zwischenberger Joseph B., Pinney Sean P. (2019). Venoarterial ECMO for Adults. Journal of the American College of Cardiology.

[4] Muller G, Flecher E, Lebreton G (2016). The ENCOURAGE mortality risk score and analysis of long-term outcomes after VA-ECMO for acute myocardial infarction with cardiogenic shock. Intensive Care Med.

[5] Truby LK, Takeda K, Mauro C (2017). Incidence and implications of left ventricular distention during venoarterial extracorporeal membrane oxygenation support. ASAIO J.

[6] Russo Juan J., Aleksova Natasha, Pitcher Ian, Couture Etienne, Parlow Simon, Faraz Mohammad, Visintini Sarah, Simard Trevor, Di Santo Pietro, Mathew Rebecca, So Derek Y., Takeda Koji, Garan A. Reshad, Karmpaliotis Dimitrios, Takayama Hiroo, Kirtane Ajay J., Hibbert Benjamin (2019). Left Ventricular Unloading During Extracorporeal Membrane Oxygenation in Patients With Cardiogenic Shock. Journal of the American College of Cardiology.

[7] Pappalardo F, Schulte C, Pieri M (2017). Concomitant implantation of Impella (R) on top of veno-arterial extracorporeal membrane oxygenation may improve survival of patients with cardiogenic shock. Eur J Heart Fail.

[8] Tehrani BN, Truesdell AG, Sherwood MW (2019). Standardized team-based care for cardiogenic shock. J Am Coll Cardiol.

