# Supplementary material for: Should ECMO be used in cardiogenic shock?
Source: Crit Care. 2019 May 16;23:174. doi: 10.1186/s13054-019-2453-2 (PMC6521371; doi:10.1186/s13054-019-2453-2)
Supplement: Related texts

# Reply: Authors’ response

**Authors:** Ludhmila Abrahao Hajjar, Jean Louis Teboul

We thank Orhan Gokalp et al. for their interest in our article. Acute myocardial infarction (AMI) is the most frequent cause of cardiogenic shock (CS) accounting for about 80% of cases [2]. The most severe cases of CS can be treated with mechanical circulatory support, as a bridge to recovery of cardiac function, or as a bridge to heart transplantation or sometimes as a bridge to another device. Veno-arterial (VA)-extracorporeal membrane oxygenation (ECMO) technique is nowadays widely recognized as an efficient assist device and easy to implant, providing high cardiac output and respiratory support [3]. Although randomized clinical trials are lacking, several nonrandomized studies suggest a survival advantage from the early use of ECMO in CS. In a study published with 138 patients, 65 (47%) patients survived to ICU discharge. However, ECMO complications occurred in 39% of the patients [4]. A common drawback of this modality is a resultant increase in left ventricular afterload. This phenomenon results in the retrograde aortic flow, which causes a marked increase in the left ventricular (LV) afterload, which might impair myocardial recovery. The consequences of this phenomenon are left ventricular distension and increase of left ventricular end-diastole pressure (LVEDP), leading to severe pulmonary edema, increased wall stress, and myocardial oxygen consumption. About 35% of ECMO patients present left ventricular distension, and 16% requires an intervention to decompress the LV [5]. A recent meta-analysis included 3997 patients, with 1696 (42%) receiving a concomitant left ventricular unloading strategy while on VA-ECMO (intra-aortic balloon pump 91.7%, percutaneous ventricular assist device 5.5%, and pulmonary vein or transseptal left atrial cannulation 2.8%). Mortality was lower in patients with (54%) versus without (65%) left ventricular unloading while on VA-ECMO (risk ratio, 0.79; *p* < 0.00001) [6]. Furthermore, recent results analyzing patients treated with concomitant VA-ECMO and Impella have shown a significantly lower in-hospital mortality and a higher rate of successful bridging to either recovery or next therapy as compared to VA-ECMO alone [7].

We do not agree with the authors that instead of VA-ECMO, it would be more appropriate to use devices such as TandemHeart or Impella, in which the left ventricle is vented in patients with a higher likelihood of recovery. We strongly believe that ECMO is the most appropriate device for severe cases of CS and that unloading the left ventricle is essential. A device such as IMPELLA might be the best option to decrease afterload and should be inserted concomitantly (ECMELLA). Moreover, as recently published, a standardized team-based approach may improve CS outcomes, increasing significantly 30-day survival from 47 to 76.6% [8]. Prompt recognition, advanced monitoring, adequate reperfusion strategies, and early implant of mechanical circulatory support are essential to improve outcomes in cardiogenic shock.
